# Impact of a content-based image retrieval system on the interpretation of chest CTs of patients with diffuse parenchymal lung disease

**DOI:** 10.1007/s00330-022-08973-3

**Published:** 2022-07-02

**Authors:** Sebastian Röhrich, Benedikt H. Heidinger, Florian Prayer, Michael Weber, Markus Krenn, Rui Zhang, Julie Sufana, Jakob Scheithe, Incifer Kanbur, Aida Korajac, Nina Pötsch, Marcus Raudner, Ali Al-Mukhtar, Barbara J. Fueger, Ruxandra-Iulia Milos, Martina Scharitzer, Georg Langs, Helmut Prosch

**Affiliations:** 1 Department of Biomedical Imaging and Image-guided Therapy, Medical University of Vienna, Währinger Gürtel 18-20, 1090 Vienna, Austria; 2 contextflow GmbH, Vienna, Austria; 3 Computational Imaging Research Lab, Department of Biomedical Imaging and Image-guided Therapy, Medical University of Vienna, Vienna, Austria

**Keywords:** Lung diseases, Interstitial, Tomography, X-ray computed, Diagnosis, Computer assisted, Artificial intelligence

## Abstract

**Objectives:**

Content-based image retrieval systems (CBIRS) are a new and potentially impactful tool for radiological reporting, but their clinical evaluation is largely missing. This study aimed at assessing the effect of CBIRS on the interpretation of chest CT scans from patients with suspected diffuse parenchymal lung disease (DPLD).

**Materials and methods:**

A total of 108 retrospectively included chest CT scans with 22 unique, clinically and/or histopathologically verified diagnoses were read by eight radiologists (four residents, four attending, median years reading chest CT scans 2.1± 0.7 and 12 ± 1.8, respectively). The radiologists read and provided the suspected diagnosis at a certified radiological workstation to simulate clinical routine. Half of the readings were done without CBIRS and half with the additional support of the CBIRS. The CBIRS retrieved the most likely of 19 lung-specific patterns from a large database of 6542 thin-section CT scans and provided relevant information (e.g., a list of potential differential diagnoses).

**Results:**

Reading time decreased by 31.3% (*p* < 0.001) despite the radiologists searching for additional information more frequently when the CBIRS was available (154 [72%] vs. 95 [43%], *p* < 0.001). There was a trend towards higher overall diagnostic accuracy (42.2% vs 34.7%, *p* = 0.083) when the CBIRS was available.

**Conclusion:**

The use of the CBIRS had a beneficial impact on the reading time of chest CT scans in cases with DPLD. In addition, both resident and attending radiologists were more likely to consult informational resources if they had access to the CBIRS. Further studies are needed to confirm the observed trend towards increased diagnostic accuracy with the use of a CBIRS in practice.

**Key Points:**

*• A content-based image retrieval system for supporting the diagnostic process of reading chest CT scans can decrease reading time by 31.3% (p < 0.001).*

*• The decrease in reading time was present despite frequent usage of the content-based image retrieval system.*

*• Additionally, a trend towards higher diagnostic accuracy was observed when using the content-based image retrieval system (42.2% vs 34.7%, p = 0.083).*

**Supplementary Information:**

The online version contains supplementary material available at 10.1007/s00330-022-08973-3.

## Introduction

Content-based image retrieval (CBIR) is an application of machine learning that attempts to alleviate the challenge of searching for digital images in large databases. In comparison to conventional search techniques that analyze text, keywords or other metadata, CBIR analyzes the “content” of the image. In general, CBIR allows the automated search of similar images to a chosen image or part of an image. As an example, this would allow to browse any accessible database for cases similar to the present case based on patterns and other imaging features. Further, this search could be narrowed by adding traditional non-imaging information, as long as this information is available, and the specific CBIR-system was trained on such data. In the radiology domain, it allows radiologists to query an image from a current study and automatically receive similar cases from the local picture archiving and communication system (PACS) or online image databases [1]. In addition to providing similar cases, some CBIR systems do also provide case-specific educational content or links to relevant online educational information linked to the case which may help the radiologists to narrow the differential diagnosis. Beyond case databases, this may accelerate the process of accessing relevant information from sources such as Radiopedia.org [2]. There, an abundance of educational cases is available, yet search is based on textual entry and not linked to the image information of a case at hand.

Thus far, research on CBIR has primarily focused on technical capabilities such as retrieval performance. However, studies evaluating the benefit of CBIR systems in a clinical setting are scarce [3–7]. A previous work assessed CBIR applications for diagnosing pulmonary pathologies in chest CTs in an experimental setting, specifically complex pathologies such as diffuse parenchymal lung diseases (DPLDs) [8–10]; however, studies adopting a practical approach towards the reading of chest CTs in a realistic setting with a wide variety of possibly rare findings that would demand considerable experience are yet lacking. This evidence of the potential impact of CBIR on the clinical routine including time savings or improved accuracy is necessary for its informed adoption. This study therefore aimed at assessing the impact of a CBIR diagnostic support system (CBIRS) on the reading time and diagnostic accuracy with the diagnosis of chest CT scans from patients with suspected DPLDs.

## Methods

### Study design

**Fig. 1:**
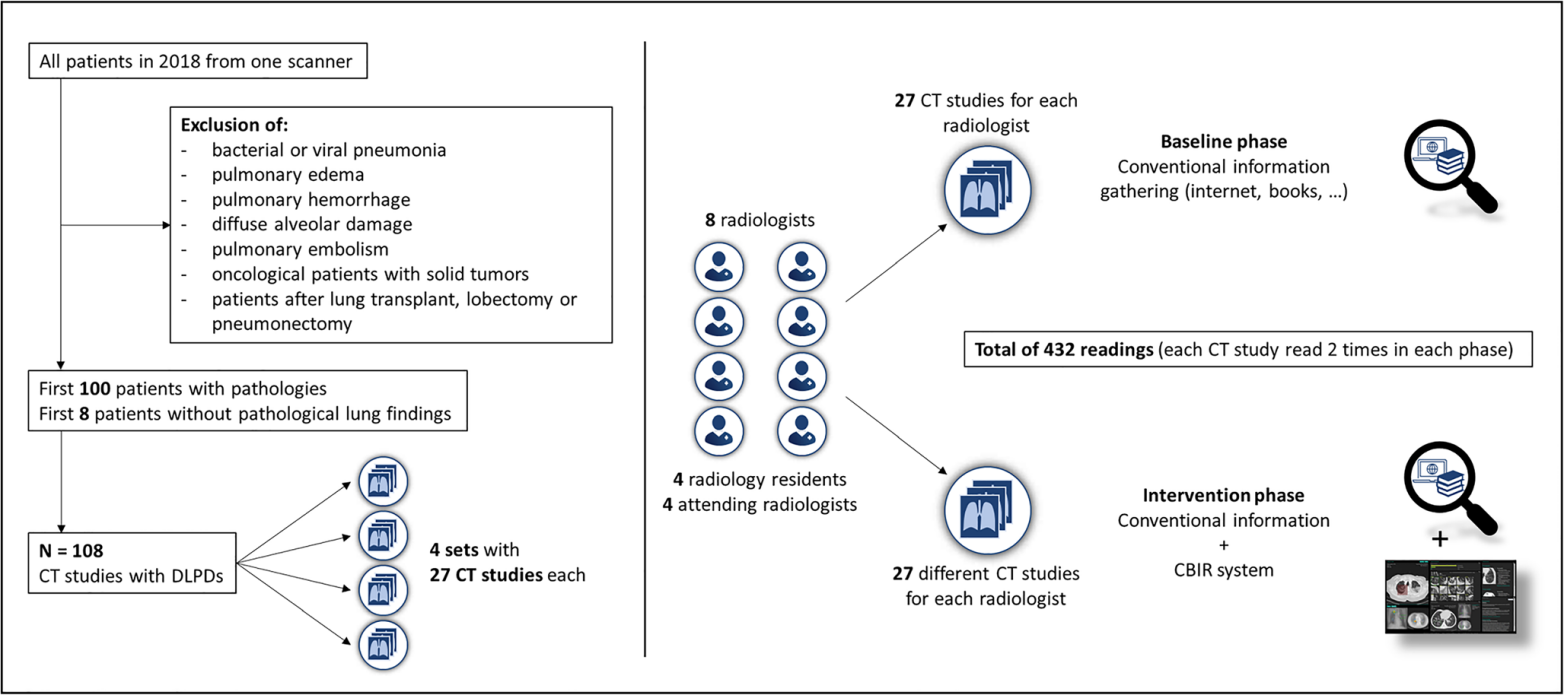
Left: exclusion and inclusion criteria. Right: distribution of cases — 108 distinct cases were distributed to 8 participants (4 junior and 4 senior radiologists), balancing out diseases between sets, where possible. Each participant read 54 distinct cases (27 during baseline and intervention phase). This way, each case was read 4 times (2 times during each phase) resulting in a total of 432 readings. Each of the 27-case sets included 2 cases without pathological lung findings. The sets were randomly assigned to the radiologists. Participants were allowed to use their own means of information gathering (internet, books, etc.) during both phases with the addition of the CBIR system as an option during the intervention phase. DLPD, diffuse parenchymal lung disease; CBIRS, content-based image retrieval system

The study was approved by the institutional review board (protocol number 1288/2018), and the need for informed consent was waived due to the retrospective nature. The study consisted of two phases: baseline and intervention. In the baseline phase, participants read chest CTs without support from the CBIRS but were allowed to use any additional sources they typically use during clinical routine. In the subsequent intervention phase, participants had access to the CBIRS while reading the chest CTs (Fig. 1).

The participants consisted of four radiology residents and four attending radiologists without sub-specialization in pulmonary imaging with a mean of 2.1 ± 0.7 and 12 ± 1.8 years of experience in reading chest CT scans, respectively; their median number of chest CT reports were 528 ± 135 and 2050 ± 1110, respectively (Table 1).

**Table 1:** Experience of participating radiologists in years and finished chest CT reports from the last 10 years

	Years experience	Chest-CT reports at baseline	Chest-CT reports at intervention	Additional chest-CT reports between baseline and intervention	Months between baseline and intervention
Radiology residents	1.7	251	736	485	14.8
	3.7	560	670	110	12.5
	1.7	496	743	247	13.3
	2.5	728	937	209	9.6
Median and mean deviation	2.1 ± 0.7	528 ± 135	739 ± 82	228 ± 111	12.9 ± 1.5
Attending radiologists	13	4175	4232	57	12.6
	10	2795	3341	546	12.1
	11	1305	1739	434	12.9
	15	1222	1433	211	12.3
Median and mean deviation	12 ± 1.8	2050 ± 1110	2540 ± 1100	323 ± 178	12.4 ± 0.3

**Fig. 2:**
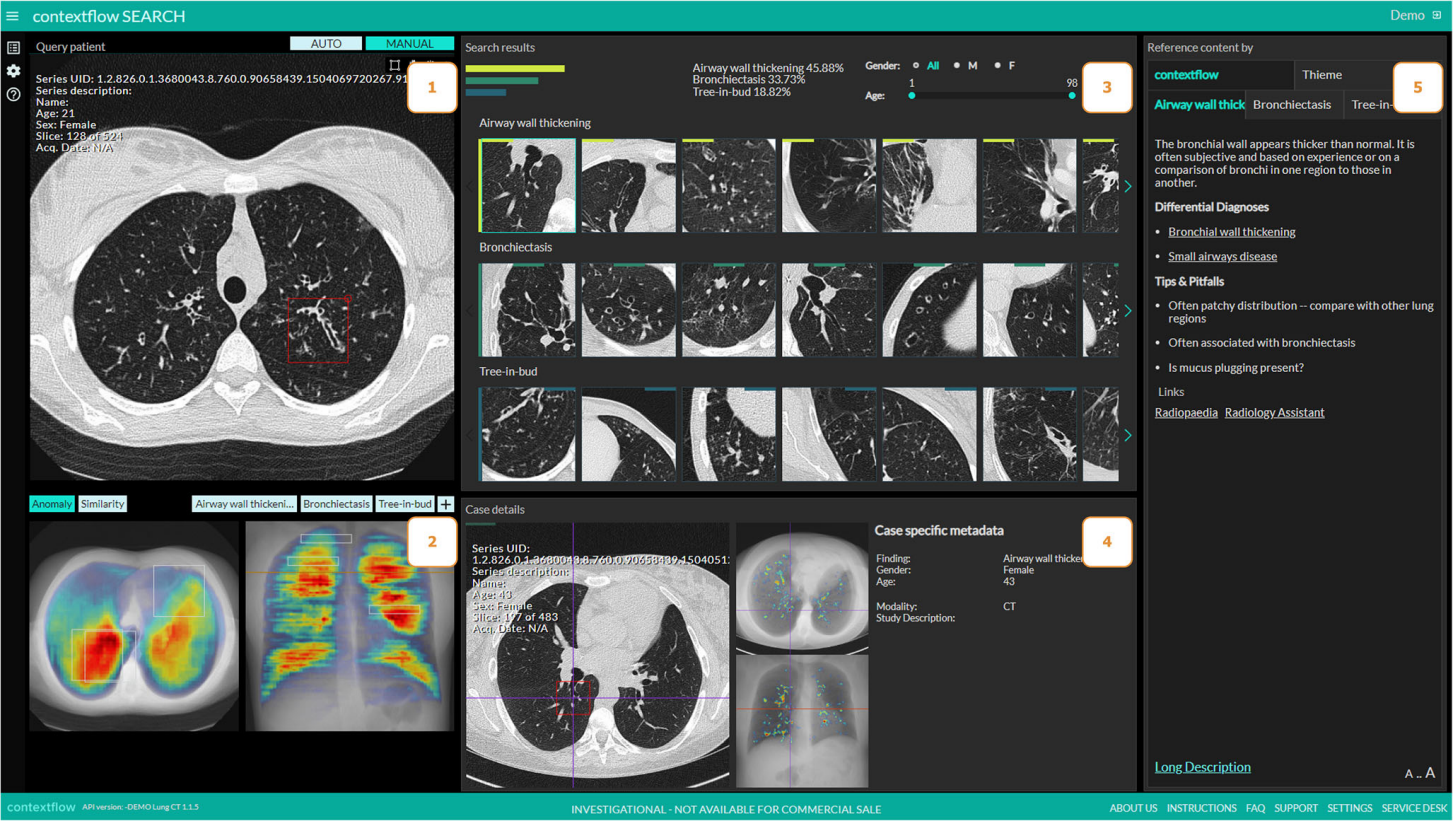
The content-based image retrieval system (CBIRS), which was used during the intervention phase, is a web application executable from the local picture archiving and communication system (PACS). (1) The radiologist initiates the search for similar cases by drawing a ROI in the current CT scan. (2) A heatmap visualizes and quantifies the distribution of one of 19 selectable lung patterns for the current scan. (3) Similar cases based on the 3 most predominant patterns in the ROI are shown arranged according to the highest lung pattern classification probability. Choosing one case leads to: (4) more detailed information from the visually similar case. (5) Content relevant to the predominant pattern is presented as a list of differential diagnoses with links to the respective Radiopaedia [2] page, tips and pitfalls for the patterns and additional in-product content for differential diagnoses

To simulate a clinical reporting situation, the assessment was performed at a dedicated clinical picture archiving and communication system (PACS) workstation. For the intervention phase, a shortcut integrated into the PACS opened the CBIRS for the respective case within 5 s. The participants could place a region of interest (ROI) within the selected CT examination. After activating the ROI-drawing mode by pressing the appropriate hotkey, a rectangular ROI of freely adjustable size can be placed within an axial image. The location is fully up to the user, and, in general, will be somewhere in or around a possibly relevant pulmonary CT-pattern. The CBIRS then identified similar cases stored in the PACS, and provided additional information related to the pattern defined by the ROI (Fig. 2).

All participants were briefed on the CBIRS’ functionality before the intervention phase and had the chance to use the CBIRS on three cases that were not part of the evaluation. Reporting was done based on the image data of the respective cases without access to previous examinations (including previous radiological reports), or non-imaging data (e.g., laboratory tests). During both phases, participants were allowed to use any source of information (e.g., online resources or books). In the intervention phase, the participants had access to the above-described CBIRS.

In both phases, every participant read 27 cases, and the time between retrieving the image data and the completion of the reading was measured for each case. Overall, every case was read four times (but never twice by the same radiologist): by one radiology resident and one attending radiologist in the baseline phase, and by a different resident and different attending radiologists in the intervention phase.

The reading time was taken by an independent observer. The same observer also noted whether the reader searched for additional information.

### Study data

The database consisted of 108 individual thin-section CT scans — 100 (92.6%) with confirmed DPLDs and 8 (7.4%) with inconspicuous pulmonary CT scans. None of the 108 cases was used for training the CBIRS. Chest CT scans performed between 1.1.2018 and 31.12.2018 on one single scanner (CT Somatom Drive, Siemens Healthineers) were included. Exclusion criteria included the following: (a) acute pulmonary pathologies; (b) patients with any kind of pulmonary surgery; (c) all inconspicuous pulmonary CTs after the first eight cases without any pathological imaging findings. Most of the indications for the 108 CT scans were either a follow-up examination in case of an already known disease or the primary CT-scan in case of a clinically suspected disease. In some cases, the CT findings were incidental, and the scan was conducted for another reason not covered by the exclusion criteria. The final database resulted in 108 cases (Table 2, Supplemental Table 1). The eight inconspicuous cases without any pulmonary pathological imaging findings served as two “healthy” cases per participant per session. Participants were told that their case sets may include “healthy” cases.

**Table 2:** Cumulative diagnoses of the evaluation cases

Number of cases	All distinct diagnoses that occurred in the study database
1	Birt-Hogg-Dubé syndrome
2	Bronchiectasis
2	Chronic obstructive pulmonary disease (COPD)
5	Chronic thromboembolic pulmonary hypertension (CTEPH)
2	Ciliary dyskinesia
5	Desquamative interstitial pneumonia (DIP)
1	Eosinophilic granulomatosis with polyangiitis (Churg-Strauss syndrome)
2	Eosinophilic pneumonia
3	Hypersensitivity pneumonitis
2	Indeterminate for usual interstitial pneumonia (UIP)
2	Lymphocytic interstitial pneumonia (LIP)
8	No pathological lung changes
23	Non-specific interstitial pneumonia (NSIP)
3	Non-classifiable interstitial lung disease
4	Organizing pneumonia (OP)
9	Probable usual interstitial pneumonia (UIP)
2	Pulmonary hypertension
1	Respiratory bronchiolitis (RB)
7	Sarcoidosis
5	Small and large airways disease
13	Usual interstitial pneumonia pattern (UIP)
6	Vasculitis
**108**	

For each case, the final diagnosis was confirmed by one sub-specialized thoracic radiologist (H.P., 20 years of experience) where available using prior and follow-up examinations, clinical symptoms, pathology and histology reports, and interdisciplinary board decisions.

### Technical specifications

All cases were reconstructed using a sharp (I70f) kernel and 1-mm slice thickness. The acquisition was done with activated automatic tube modulation, 90, 100, or 140 kV and either without contrast agent (*n* = 58), during a pulmonary arterial phase (*n* = 15; total of 50 ml of 400 mg iodine per ml contrast agent with a threshold trigger at 115 HU and a 5-s delay) or during a venous phase (*n* = 35; total of 70 ml of 400 mg iodine per ml contrast agent after a 50-s delay).

### CBIR system

A research prototype of contextflow SEARCH Lung CT (contextflow GmbH) was used as the CBIRS. After manually marking a region of interest (ROI), it searches a large database of 6542 anonymized thin-section CT scans (2528 female, 4014 male, ages 5 to 100 years, acquired over 2.5 years) for similar imaging patterns. Each of the CTs in this training dataset included expert labels for 19 lung-specific patterns. A content-specific similarity function based on these expert-labeled data was developed and used to compare image patterns. For any marked region of interest, the CBIRS provides the three most likely disease patterns present. For every pattern class, it additionally provides the cases that most closely resemble the marked ROI in the current scan (Fig. 2).

The image retrieval algorithm searches for image patches similar to the region of interest image patch (ROI) marked by the user in the current case. This ROI serves as a query, and retrieval identifies the most similar patches in a large pre-curated database of patches extracted from lung CTs. We created this search database of over 5,000,000,000 such patches, involving two steps: (1) learning a similarity function that puts patches of the same disease pattern in close proximity, so that the nearest neighbors of a newly observed patch have the same disease pattern, and (2) creating an index, so that during actual search the comparison of the ROI with the database patches can be performed rapidly (within 1 s).

The similarity function is learned from training data consisting of thin-section CTs. Lungs are segmented and from within the lung region patches are extracted. The entirety of these patches is used to learn an embedding of patches into an embedding space. There, each patch is represented by a coordinate, so that the Euclidean distance between patch representatives can serve as a similarity function. The embedding is performed by a feed forward convolutional neural network, its input is the image patch, and the output the coordinates of the representative in the embedding space.

A direct comparison of a query patch with all database patches calculating billions of distances during the interactive use is not feasible. Instead, an index of the coordinates in the embedding space is built to accelerate retrieval to less than 1 s. A separate index is built for each disease pattern class. To this end the embedding is subdivided in a hierarchical fashion to build a two-level data structure, containing information on the coarse and fine location of individual patches in the embedding space.

During search, first the ROI is embedded using the feed forward network, the three most likely pattern classes are estimated, and then search for the most similar patches is conducted in the hierarchical index for each pattern class.

The 19 lung pattern classes are as follows: airway wall thickening, atelectasis, bronchiectasis, bulla, consolidation, cysts, effusion, emphysema, ground-glass opacities, honeycombing, mass, mosaic attenuation pattern, nodular pattern, nodule, pneumothorax, pulmonary cavity, reticular pattern, tree in bud, non-specific (includes inconspicuous lung regions).

### Statistical evaluation

All statistical computations were performed using SPSS v26.0 (version 26.0, IBM Corp.). *p*-values equal or below 0.05 are considered statistically significant. A sample size calculation was conducted to establish the necessary number of cases. For the variable “correct diagnosis,” a power of 80%, two-sided alpha of 5% and 20% increase in correct diagnoses were chosen. Under the assumption of 60% diagnostic accuracy for different DPLDs [11], this equals an increase of 12 percentage points, resulting in 83 necessary cases when analyzed with McNemar’s test. Rounded up, we included 100 cases with lung pathologies. Continuous variables were tested for normal distribution using Shapiro-Wilk test and via visual assessment per subgroup and are displayed either as mean ± standard deviation or as median [interquartile range] as appropriate. To evaluate differences in time-to-diagnosis and correct diagnoses, three-way linear mixed models with an unstructured covariance matrix were used. The three factors were as follows: “phase” (baseline vs. intervention), “reader” (eight different readers subdivided into two groups of experience), and “if the participant looked for additional information” (yes vs. no). All main effects and two-way interactions were included. For post hoc analyses, Bonferroni multiplicity corrections were assessed.

## Results

**Fig. 3:**
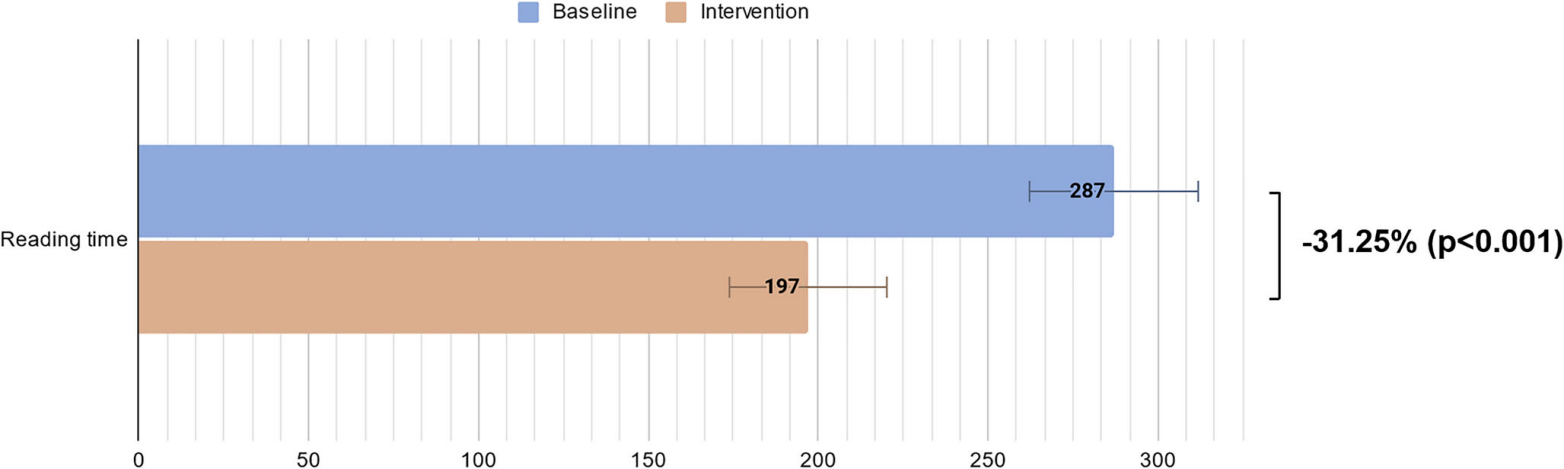
Modeled overall reading time during baseline and intervention phase in seconds with 95% confidence intervals

Modeled overall time used per case was reduced by 31.3% when using the CBIRS (*p* < 0.001), controlling for individual participants, experience level, and whether they looked for information (Fig. 3). The reduction in time taken per case from baseline to intervention was more distinct for cases where the participants looked for information compared to where they did not (110 vs 39 s saved, *p* = 0.002) (Table 3).

**Table 3:** Overview of reading time and accuracy of diagnoses depending on study phase, if the participants looked for information (including both conventional ways and the CBIR system) as well as the participant experience (junior or senior radiologist). Two readings during the intervention phase had to be excluded due to technical difficulties, resulting in a total of 214 cases for this phase instead of 216

Phase	Looked for information?	Experience	Reading time (sec)	Correct diagnoses	Number
Baseline	No	Resident	166 ± 82	54.7%	53
		Attending	201 ± 174	51.5%	68
		**Total**	**186 ± 142**	**52.9%**	**121**
	Yes	Resident	438 ± 250	41.8%	55
		Attending	344 ± 156	25.0%	40
		**Total**	**398 ± 220**	**34.7%**	**95**
	**Total**	Resident	305 ± 231	48.1%	108
		Attending	254 ± 180	41.7%	108
		**Total**	**279 ± 209**	**44.9%**	**216**
Intervention	No	Resident	116 ± 65	56.8%	44
		Attending	111 ± 68	62.5%	16
		**Total**	**115 ± 65**	**58.3%**	**60**
	Yes	Resident	321 ± 189	42.9%	63
		Attending	224 ± 123	41.8%	91
		**Total**	**264 ± 160**	**42.2%**	**154**
	**Total**	Resident	237 ± 181	48.6%	107
		Attending	207 ± 123	44.9%	107
		**Total**	**222 ± 156**	**46.7%**	**214**
Total	No	Resident	143 ± 79	55.7%	97
		Attending	184 ± 163	53.6%	84
		**Total**	**162 ± 126**	**54.7%**	**181**
	Yes	Resident	376 ± 226	42.4%	118
		Attending	261 ± 145	36.6%	131
		**Total**	**315 ± 196**	**39.4%**	**249**
	**Total**	Resident	271 ± 210	48.4%	215
		Attending	231 ± 156	43.3%	215
		**Total**	**251 ± 186**	**45.8%**	**430**

Overall diagnostic accuracy was higher in the interventional phase compared to the baseline phase without reaching statistical significance (42.2% vs 34.7%, *p* = 0.083).

Participants used additional information (e.g., internet searches) more frequently during the intervention phase compared to the baseline phase (154 [72%] vs 95 [43%], *p* < 0.001). Of the 154 information searches, 108 (70.1%) were conducted solely with the CBIRS and 46 (29.9%) with the CBIRS plus traditional means.

Despite searching for additional information more frequently, both the radiology residents and attending radiologists showed a decrease in reading time when the CBIRS was available, and there was a tendency towards a stronger decrease in reading time for senior radiologists (27% vs 35%, *p* = 0.078).

## Discussion

In this study we found that the integration of a CBIRS into the clinical workflow of radiologists reading chest CTs resulted in a significant decrease in average reading time.

The main hypothesized advantage of the CBIRS is the streamlined provision of relevant information for the case the radiologist is currently reporting on. One of the most acclaimed informational sources is Radiopaedia, providing over 45,000 cases where quality and actuality are ensured by the editorial board consisting of radiological experts. Several studies have shown Radiopaedia to be the top or one of the most used informational sources [12, 13]. Furthermore, an educational benefit was demonstrated for integrating Radiopaedia-based training in a medical curriculum [14], underlining the potential long-term on radiological experience. Similar to how contemporary search engines work, we aimed at closing the gap between the image and the information that is necessary to provide the correct diagnosis by providing both no-click results after the search and a direct link to the most relevant informational site (i.e., the corresponding Radiopaedia article). Indeed, both for radiology residents and attending radiologists, the decrease in average reading time was more pronounced when they looked for additional information. This is particularly relevant as the participants used additional information more frequently during the intervention phase compared to the baseline phase (72% vs. 43%, *p* < 0.001). The CBIRS was used in all of these instances (70.1% of the information searches were conducted solely with the CBIRS and 29.9% with the CBIRS plus traditional means). This indicates a more focused and efficient search for information when the CBIRS is available.

Although participants were given different cases for each phase, reading times may still have been affected by familiarity with the study process and the individual drive to finish the readings. However, even after adjusting for alternative factors in our model, including phase, reader type, and additional information search, the overall 31.3% decrease in reading time remained and was concurrent with a trend towards higher diagnostic accuracy when the CBIRS was available (42.2% vs 34.7%, *p* = 0.083).

Reporting was done based on the image data of the respective cases without access to previous examinations (including previous radiological reports), or non-imaging data (e.g., laboratory tests). This limits the highest achievable accuracy for complex cases such as in interstitial lung disease. Although the participants may not have had a confident diagnosis available, they still had to commit to giving one, resulting in the shown overall diagnostic accuracy (which may be quite low even for experienced thoracic specialists as reported by Walsh et. al [15]).

Previous studies assessing CBIRS in lung imaging have demonstrated significant improvements in diagnostic accuracy: 13% for pulmonary nodules [9] and 15–62% for various disease patterns and diagnoses [8, 10, 16]. However, most of these studies were met with significant limitations, including application solely for pulmonary nodules, low number of test cases, and inclusion of individual images (rather than volumes) in the training data. This study aimed to simulate clinical routine, leaving participants the decision whether and how to search for information (including all options in both phases). This design avoids possible biases and more closely reflects routine workflow, in contrast to *forcing* radiologists to use information search. While previous studies report a benefit mainly for less experienced participants [8], this study showed significant time savings both for junior and senior radiologists. A similar study showed the feasibility of improving diagnostic accuracy while investigating a CBIR for aiding the diagnosis of ILD [16]. The CBIR applied in the referenced paper had an additional query database with labeled diagnoses for the query cases, which was not in place yet for the CBIR used during our study. For further studies we suggest adding well-curated diagnosis labels to the query database.

As we encountered delays as a result of the ongoing SARS-CoV-2 pandemic, time between the two phases ranged from 9 and 15 months for individual participants. Thus, increasing experience between phases constitutes another potential bias. However, the results for radiology residents and attending radiologists were similar, even though the latter had a markedly less pronounced relative increase in years of professional experience and finished reports (Table 1).

In conclusion, the presence of a CBIRS when reading chest CTs for patients with suspected DPLD resulted in an overall 31% decrease in reading time despite participants searching for additional information more frequently. This is of particular interest, as a recent study [17] reports that about 86% of studies using AI could lead to an increase in radiologists’ workload. In addition, we noted a non-significant trend towards improved diagnostic accuracy. These findings hold true for both junior and senior radiologists. Such optimistic outcomes warrant additional research of CBIRS in clinical settings.

## Supplementary information

ESM 1 (DOCX 21 kb)

## Abbreviations

CBIRS: Content-based image retrieval system
CT: Computed tomography
DPLD: Diffuse parenchymal lung disease
PACS: Picture archiving and communication system
ROI: Region of interest

## Declarations

### Guarantor

The scientific guarantor of this publication is Dr. Georg Langs.

### Statistics and biometry

One of the authors has significant statistical expertise.

### Methodology

• retrospective

• performed at one institution
