# Supplementary material for: Impact of a content-based image retrieval system on the interpretation of chest CTs of patients with diffuse parenchymal lung disease
Source: Eur Radiol. 2022 Jul 2;33(1):360–7. doi: 10.1007/s00330-022-08973-3 (PMC9755072; doi:10.1007/s00330-022-08973-3)
Supplement: Supplementary file 1 — (DOCX 21 kb) [file 330_2022_8973_MOESM1_ESM.docx]

# Supplemental Materials

| **Case ID** | **Age in years** | **Diagnosis** |
| --- | --- | --- |
| 1 | 74 | Usual interstitial pneumonia pattern (UIP) |
| 2 | 41 | Non specific interstitial pneumonia (NSIP) |
| 3 | 68 | Chronic thromboembolic pulmonary hypertension (CTEPH) |
| 4 | 34 | Usual interstitial pneumonia pattern (UIP) |
| 5 | 85 | Small and large airways disease |
| 6 | 45 | Vasculitis |
| 7 | 79 | Usual interstitial pneumonia pattern (UIP) |
| 8 | 69 | Probable usual interstitial pneumonia (UIP) |
| 9 | 54 | Vasculitis |
| 10 | 67 | Non specific interstitial pneumonia (NSIP) |
| 11 | 61 | Non specific interstitial pneumonia (NSIP) |
| 12 | 56 | Non specific interstitial pneumonia (NSIP) |
| 13 | 62 | Desquamative interstitial pneumonia (DIP) |
| 14 | 56 | Non specific interstitial pneumonia (NSIP) |
| 15 | 25 | Non specific interstitial pneumonia (NSIP) |
| 16 | 34 | Usual interstitial pneumonia pattern (UIP) |
| 17 | 45 | Desquamative interstitial pneumonia (DIP) |
| 18 | 56 | Hypersensitivity pneumonitis |
| 19 | 75 | Probable usual interstitial pneumonia (UIP) |
| 20 | 32 | Vasculitis |
| 21 | 81 | Non specific interstitial pneumonia (NSIP) |
| 22 | 17 | Vasculitis |
| 23 | 39 | Non specific interstitial pneumonia (NSIP) |
| 24 | 71 | Usual interstitial pneumonia pattern (UIP) |
| 25 | 36 | Eosinophilic pneumonia |
| 26 | 45 | Sarcoidosis |
| 27 | 74 | Desquamative interstitial pneumonia (DIP) |
| 28 | 74 | Probable usual interstitial pneumonia (UIP) |
| 29 | 72 | Chronic thromboembolic pulmonary hypertension (CTEPH) |
| 30 | 87 | Probable usual interstitial pneumonia (UIP) |
| 31 | 54 | Non-classifiable interstitial lung disease |
| 32 | 74 | Eosinophilic pneumonia |
| 33 | 78 | Usual interstitial pneumonia pattern (UIP) |
| 34 | 55 | Non specific interstitial pneumonia (NSIP) |
| 35 | 82 | Probable usual interstitial pneumonia (UIP) |
| 36 | 17 | Eosinophilic granulomatosis with polyangiitis (Churg-Strauss-Syndrome) |
| 37 | 76 | Birt-Hogg-Dubé-Syndrome |
| 38 | 54 | Non specific interstitial pneumonia (NSIP) |
| 39 | 49 | Non specific interstitial pneumonia (NSIP) |
| 40 | 46 | Sarcoidosis |
| 41 | 64 | Sarcoidosis |
| 42 | 52 | Lymphocytic interstitial pneumonia (LIP) |
| 43 | 69 | Sarcoidosis |
| 44 | 54 | Small and large airways disease |
| 45 | 65 | Usual interstitial pneumonia pattern (UIP) |
| 46 | 36 | Pulmonary hypertension |
| 47 | 62 | Non specific interstitial pneumonia (NSIP) |
| 48 | 73 | Sarcoidosis |
| 49 | 63 | Chronic obstructive pulmonary disease (COPD) |
| 50 | 32 | Pulmonary hypertension |
| 51 | 64 | Non specific interstitial pneumonia (NSIP) |
| 52 | 44 | Bronchiectasis |
| 53 | 44 | Indeterminate for usual interstitial pneumonia (UIP) |
| 54 | 77 | Probable usual interstitial pneumonia (UIP) |
| 55 | 81 | Non-classifiable interstitial lung disease |
| 56 | 34 | Non specific interstitial pneumonia (NSIP) |
| 57 | 66 | Usual interstitial pneumonia pattern (UIP) |
| 58 | 57 | Hypersensitivity pneumonitis |
| 59 | 31 | Non specific interstitial pneumonia (NSIP) |
| 60 | 63 | Organizing pneumonia (OP) |
| 61 | 74 | Usual interstitial pneumonia pattern (UIP) |
| 62 | 62 | Chronic thromboembolic pulmonary hypertension (CTEPH) |
| 63 | 61 | Small and large airways disease |
| 64 | 83 | Probable usual interstitial pneumonia (UIP) |
| 65 | 53 | Organizing pneumonia (OP) |
| 66 | 72 | Usual interstitial pneumonia pattern (UIP) |
| 67 | 76 | Indeterminate for Usual Interstitial Pneumonia (UIP) |
| 68 | 75 | Usual interstitial pneumonia pattern (UIP) |
| 69 | 71 | Usual interstitial pneumonia pattern (UIP) |
| 70 | 84 | Sarcoidosis |
| 71 | 70 | Non specific interstitial pneumonia (NSIP) |
| 72 | 54 | Small and large airways disease |
| 73 | 74 | Vasculitis |
| 74 | 50 | Sarcoidosis |
| 75 | 69 | Vasculitis |
| 76 | 77 | Probable usual interstitial pneumonia (UIP) |
| 77 | 62 | Non-classifiable interstitial lung disease |
| 78 | 65 | Non specific interstitial pneumonia (NSIP) |
| 79 | 78 | Usual interstitial pneumonia pattern (UIP) |
| 80 | 72 | Non specific interstitial pneumonia (NSIP) |
| 81 | 71 | Chronic obstructive pulmonary disease (COPD) |
| 82 | 75 | Non specific interstitial pneumonia (NSIP) |
| 83 | 78 | Hypersensitivity pneumonitis |
| 84 | 27 | Ciliary dyskinesia |
| 85 | 49 | Non specific interstitial pneumonia (NSIP) |
| 86 | 61 | Lymphocytic interstitial pneumonia (LIP) |
| 87 | 49 | Organizing pneumonia (OP) |
| 88 | 76 | Chronic thromboembolic pulmonary hypertension (CTEPH) |
| 89 | 74 | Desquamative interstitial pneumonia (DIP) |
| 90 | 54 | Non specific interstitial pneumonia (NSIP) |
| 91 | 46 | Small and large airways disease |
| 92 | 71 | Organizing pneumonia (OP) |
| 93 | 77 | Chronic thromboembolic pulmonary hypertension (CTEPH) |
| 94 | 72 | Bronchiectasis |
| 95 | 75 | Non specific interstitial pneumonia (NSIP) |
| 96 | 77 | Non specific interstitial pneumonia (NSIP) |
| 97 | 51 | Ciliary dyskinesia |
| 98 | 71 | Desquamative interstitial pneumonia (DIP) |
| 99 | 79 | Probable usual interstitial pneumonia (UIP) |
| 100 | 54 | Respiratory bronchiolitis (RB) |
| 101 | 32 | No pathological lung changes |
| 102 | 42 | No pathological lung changes |
| 103 | 57 | No pathological lung changes |
| 104 | 22 | No pathological lung changes |
| 105 | 23 | No pathological lung changes |
| 106 | 61 | No pathological lung changes |
| 107 | 44 | No pathological lung changes |
| 108 | 53 | No pathological lung changes |

**Supplemental table 1 -** Age and diagnoses of the patients from the cases the participating radiologists had to read. Mean age of patients was 60 ± 17 years.
